# Poly[[tris­(μ_2_-4,4′-bipyridine *N*,*N*′-di­oxide)hexa­nitratodigadolinium(III)] dichloro­methane disolvate]

**DOI:** 10.1107/S1600536810033258

**Published:** 2010-08-21

**Authors:** Adam J. Dillner, Cassandra P. Lilly, Jacqueline M. Knaust

**Affiliations:** a Allegheny College, 520 North Main St., Meadville, PA 16335, USA

## Abstract

The title one-dimensional coordination network, {[Gd_2_(NO_3_)_6_(C_10_H_8_N_2_O_2_)_3_]·2CH_2_Cl_2_}_*n*_, is isostructural with the previously reported Tb and Tl coordination networks and to its Eu analog. The Gd^III^ cation is coordinated in a distorted tricapped trigonal-prismatic fashion by nine O atoms from three bridging 4,4′-bipyridine *N*,*N*′-dioxide ligands and three chelating nitrate anions. None of the atoms lie on a special position, but there is an inversion center located between the rings of one of the ligands. The network topology is ladder-like, and each ladder inter­acts with six neighboring ladders through C—H⋯O hydrogen bonds. The packing motif of the ladders allows for the formation of channels that run parallel to the *a* axis; these channels are filled with CH_2_Cl_2_ solvent mol­ecules that inter­act with the ladders through C—H⋯O hydrogen bonds

## Related literature

For the isostructural Tb and Tl, coordination networks, see: Long *et al.* (2002 ▶); Moitsheki *et al.* (2006 ▶). For the isostructural Eu coordination network and detailed background to this study, see: Dillner *et al.* (2010 ▶).
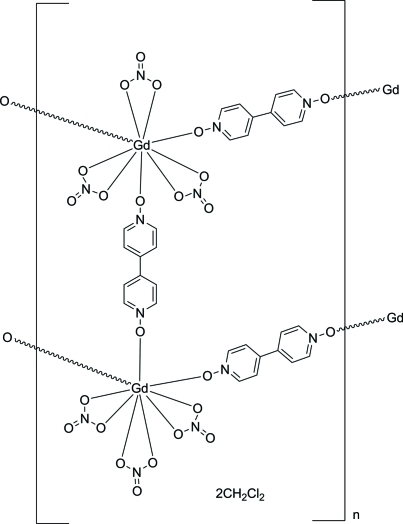


## Experimental

### 

#### Crystal data

[Gd_2_(NO_3_)_6_(C_10_H_8_N_2_O_2_)_3_]·2CH_2_Cl_2_
*M* *_r_* = 1420.96
Triclinic, 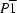

*a* = 7.9917 (5) Å
*b* = 11.5668 (7) Å
*c* = 13.0347 (8) Å
α = 86.059 (1)°
β = 80.134 (1)°
γ = 78.255 (1)°
*V* = 1161.52 (12) Å^3^
*Z* = 1
Mo *K*α radiation
μ = 3.16 mm^−1^
*T* = 100 K
0.51 × 0.48 × 0.25 mm

#### Data collection

Bruker SMART APEX CCD diffractometer
Absorption correction: multi-scan (*SADABS*; Bruker, 2009 ▶) *T*_min_ = 0.529, *T*_max_ = 0.746
13791 measured reflections
6990 independent reflections
6776 reflections with *I* > 2σ(*I*)
*R*_int_ = 0.019

#### Refinement

*R*[*F* ^2^ > 2σ(*F* ^2^)] = 0.020
*wR*(*F* ^2^) = 0.051
*S* = 1.06
6990 reflections
334 parameters
H-atom parameters constrained
Δρ_max_ = 1.34 e Å^−3^
Δρ_min_ = −1.26 e Å^−3^

### 

Data collection: *APEX2* (Bruker, 2009 ▶); cell refinement: *SAINT* (Bruker, 2009 ▶); data reduction: *SAINT*; program(s) used to solve structure: *SHELXS97* (Sheldrick, 2008 ▶); program(s) used to refine structure: *SHELXL97* (Sheldrick, 2008 ▶); molecular graphics: *X-SEED* (Barbour, 2001 ▶); software used to prepare material for publication: *X-SEED*.

## Supplementary Material

Crystal structure: contains datablocks I, global. DOI: 10.1107/S1600536810033258/zl2303sup1.cif

Structure factors: contains datablocks I. DOI: 10.1107/S1600536810033258/zl2303Isup2.hkl

Additional supplementary materials:  crystallographic information; 3D view; checkCIF report

## Figures and Tables

**Table 1:** Hydrogen-bond geometry (Å, °)

*D*—H⋯*A*	*D*—H	H⋯*A*	*D*⋯*A*	*D*—H⋯*A*
C5—H5⋯O7^i^	0.95	2.41	3.082 (2)	127
C9—H9⋯O9^ii^	0.95	2.57	3.287 (2)	132
C12—H12⋯O2^iii^	0.95	2.43	3.300 (2)	152
C16—H16*B*⋯O12^ii^	0.99	2.43	3.246 (3)	139
C16—H16*A*⋯O8	0.99	2.56	3.302 (3)	132
C16—H16*A*⋯O9	0.99	2.50	3.084 (3)	117

Symmetry codes: (i) 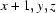; (ii) 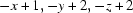; (iii) 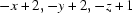.

**Fig. 1.**
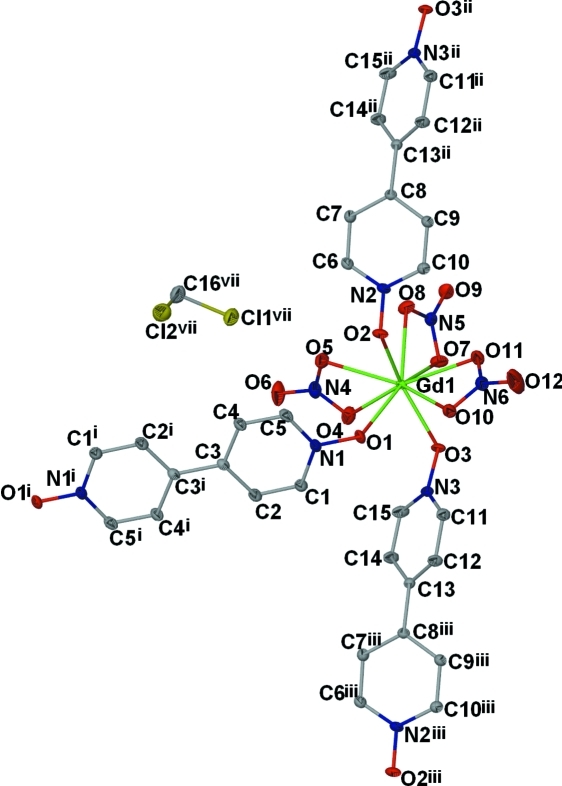
The coordination environment of the Gd+3 cation in the title compound with atom labels and 50% probability displacement ellipsoids. Hydrogen atoms have been omitted for clarity. Color scheme: Gd: green, C: grey, N: blue, O: red, Cl: yellow. Symmetry codes: (i) -x+3, -y+1, -z+1; (ii) x, y, z+1; (iii) x, y, z-1; (vii) -x+2, -y+1, z+2.

## supplementary crystallographic information

### Comment

The description of the structure of the title compound is part of a set of consecutive papers on one-dimensional ladder-like coordination networks of the type [Ln_2_(NO_3_)_6_(C_10_H_8_N_2_O_2_)_3_]_n_, with Ln = Eu (Dillner *et al.*, 2010) and Gd (this publication), respectively. Both compounds are also isostructural to the previously reported Tb and Tl, coordination networks (Long *et al.*, 2002 and Moitsheki *et al.*, 2006). The background to this study is given in Dillner *et al.* (2010).

### Experimental

Gd(NO_3_)_3_ (0.051 g 0.15 mmol) was placed in the bottom of a test tube and covered with CH_2_Cl_2_ (5 ml). *4,4*'-bipyridine-*N,N*'-dioxide^.^H_2_O (0.0376 g, 0.182 mmol) was dissolved in methanol (8 ml), and this solution was layered over the CH_2_Cl_2_. The two solutions were allowed to slowly mix. Over a period of several weeks the Gd(NO_3_)_3_ dissolved, and yellow plate-like crystals of the title compound formed.

### Refinement

All H atoms were positioned geometrically and refined using a riding model with C—H = 0.95 Å and with *U_iso_*(H) = 1.2 times *U*_eq_(C).

### Crystal data

**Table:**

[Gd_2_(NO_3_)_6_(C_10_H_8_N_2_O_2_)_3_]·2CH_2_Cl_2_	*Z* = 1
*M**_r_* = 1420.96	*F*(000) = 692
Triclinic, *P*1	*D*_x_ = 2.031 Mg m^−^^3^
Hall symbol: -P 1	Mo *K*α radiation, λ = 0.71073 Å
*a* = 7.9917 (5) Å	Cell parameters from 9995 reflections
*b* = 11.5668 (7) Å	θ = 2.4–31.4°
*c* = 13.0347 (8) Å	µ = 3.16 mm^−^^1^
α = 86.059 (1)°	*T* = 100 K
β = 80.134 (1)°	Plate, yellow
γ = 78.255 (1)°	0.51 × 0.48 × 0.25 mm
*V* = 1161.52 (12) Å^3^	

### Data collection

**Table:**

Bruker SMART APEX CCD diffractometer	6990 independent reflections
Radiation source: fine-focus sealed tube	6776 reflections with *I* > 2σ(*I*)
graphite	*R*_int_ = 0.019
ω scans	θ_max_ = 31.5°, θ_min_ = 1.6°
Absorption correction: multi-scan (*SADABS*; Bruker, 2009)	*h* = −11→11
*T*_min_ = 0.529, *T*_max_ = 0.746	*k* = −16→16
13791 measured reflections	*l* = −19→19

### Refinement

**Table:**

Refinement on *F*^2^	Primary atom site location: structure-invariant direct methods
Least-squares matrix: full	Secondary atom site location: difference Fourier map
*R*[*F*^2^ > 2σ(*F*^2^)] = 0.020	Hydrogen site location: inferred from neighbouring sites
*wR*(*F*^2^) = 0.051	H-atom parameters constrained
*S* = 1.06	*w* = 1/[σ^2^(*F*_o_^2^) + (0.0269*P*)^2^ + 0.7108*P*] where *P* = (*F*_o_^2^ + 2*F*_c_^2^)/3
6990 reflections	(Δ/σ)_max_ = 0.003
334 parameters	Δρ_max_ = 1.34 e Å^−^^3^
0 restraints	Δρ_min_ = −1.26 e Å^−^^3^

### Special details

**Table:**

Geometry. All esds (except the esd in the dihedral angle between two l.s. planes) are estimated using the full covariance matrix. The cell esds are taken into account individually in the estimation of esds in distances, angles and torsion angles; correlations between esds in cell parameters are only used when they are defined by crystal symmetry. An approximate (isotropic) treatment of cell esds is used for estimating esds involving l.s. planes.
Refinement. Refinement of F^2^ against ALL reflections. The weighted R-factor wR and goodness of fit S are based on F^2^, conventional R-factors R are based on F, with F set to zero for negative F^2^. The threshold expression of F^2^ > 2sigma(F^2^) is used only for calculating R-factors(gt) etc. and is not relevant to the choice of reflections for refinement. R-factors based on F^2^ are statistically about twice as large as those based on F, and R- factors based on ALL data will be even larger.

### Fractional atomic coordinates and isotropic or equivalent isotropic displacement parameters (Å^2^)

**Table:**

	*x*	*y*	*z*	*U*_iso_*/*U*_eq_	
Gd1	0.777386 (9)	0.833312 (7)	0.717678 (6)	0.01052 (3)	
O1	1.02444 (16)	0.82720 (11)	0.59321 (10)	0.0154 (2)	
O2	0.95666 (17)	0.87415 (12)	0.83066 (9)	0.0156 (2)	
O3	0.62864 (17)	0.87372 (12)	0.57713 (9)	0.0165 (2)	
O4	0.80192 (19)	0.63739 (12)	0.64108 (12)	0.0228 (3)	
O5	0.95223 (19)	0.63785 (12)	0.76314 (11)	0.0215 (3)	
O6	0.9729 (2)	0.47510 (14)	0.68376 (16)	0.0329 (4)	
O7	0.48223 (18)	0.79088 (13)	0.77573 (10)	0.0200 (3)	
O8	0.64333 (17)	0.77345 (13)	0.89476 (11)	0.0200 (3)	
O9	0.37413 (18)	0.75493 (13)	0.93691 (11)	0.0224 (3)	
O10	0.80809 (18)	1.04012 (12)	0.66218 (10)	0.0186 (3)	
O11	0.59902 (18)	1.01912 (12)	0.78729 (11)	0.0191 (3)	
O12	0.6429 (3)	1.19502 (15)	0.73675 (17)	0.0438 (5)	
N1	1.15539 (19)	0.73739 (13)	0.56764 (11)	0.0133 (3)	
N2	0.91991 (19)	0.86889 (13)	0.93448 (11)	0.0126 (3)	
N3	0.69504 (19)	0.86834 (13)	0.47630 (11)	0.0132 (3)	
N4	0.9111 (2)	0.57955 (14)	0.69595 (14)	0.0191 (3)	
N5	0.49582 (19)	0.77173 (13)	0.87144 (12)	0.0149 (3)	
N6	0.6826 (2)	1.08859 (15)	0.72846 (13)	0.0201 (3)	
C1	1.1729 (3)	0.68680 (17)	0.47533 (15)	0.0200 (4)	
H1	1.0924	0.7154	0.4293	0.024*	
C2	1.3074 (3)	0.59355 (18)	0.44774 (15)	0.0204 (4)	
H2	1.3185	0.5581	0.3827	0.024*	
C3	1.4277 (2)	0.55043 (15)	0.51390 (13)	0.0129 (3)	
C4	1.4065 (2)	0.60763 (17)	0.60766 (14)	0.0177 (3)	
H4	1.4869	0.5823	0.6544	0.021*	
C5	1.2700 (2)	0.70055 (17)	0.63297 (14)	0.0179 (3)	
H5	1.2569	0.7387	0.6969	0.021*	
C6	0.9834 (2)	0.76999 (16)	0.98742 (14)	0.0154 (3)	
H6	1.0519	0.7040	0.9506	0.018*	
C7	0.9492 (2)	0.76424 (15)	1.09522 (14)	0.0153 (3)	
H7	0.9932	0.6941	1.1322	0.018*	
C8	0.8502 (2)	0.86129 (15)	1.14956 (13)	0.0126 (3)	
C9	0.7879 (2)	0.96209 (15)	1.09168 (13)	0.0147 (3)	
H9	0.7208	1.0298	1.1266	0.018*	
C10	0.8227 (2)	0.96446 (15)	0.98455 (13)	0.0149 (3)	
H10	0.7785	1.0331	0.9458	0.018*	
C11	0.7464 (2)	0.96300 (15)	0.42487 (14)	0.0158 (3)	
H11	0.7456	1.0312	0.4617	0.019*	
C12	0.8003 (2)	0.96117 (16)	0.31859 (13)	0.0158 (3)	
H12	0.8341	1.0289	0.2822	0.019*	
C13	0.8053 (2)	0.86070 (15)	0.26447 (13)	0.0125 (3)	
C14	0.7610 (3)	0.76171 (16)	0.32125 (14)	0.0182 (3)	
H14	0.7694	0.6903	0.2871	0.022*	
C15	0.7051 (3)	0.76792 (17)	0.42680 (14)	0.0192 (3)	
H15	0.6733	0.7008	0.4653	0.023*	
C16	0.5603 (3)	0.60138 (19)	1.10231 (18)	0.0274 (4)	
H16A	0.5816	0.6058	1.0252	0.033*	
H16B	0.5414	0.6826	1.1273	0.033*	
Cl1	0.74334 (7)	0.51440 (4)	1.14775 (4)	0.02581 (10)	
Cl2	0.37217 (7)	0.54128 (6)	1.14632 (5)	0.03300 (12)	

### Atomic displacement parameters (Å^2^)

**Table:**

	*U*^11^	*U*^22^	*U*^33^	*U*^12^	*U*^13^	*U*^23^
Gd1	0.01086 (4)	0.01200 (4)	0.00842 (4)	−0.00199 (3)	−0.00084 (3)	−0.00099 (3)
O1	0.0135 (6)	0.0135 (5)	0.0165 (6)	0.0004 (4)	0.0023 (4)	−0.0032 (4)
O2	0.0171 (6)	0.0228 (6)	0.0077 (5)	−0.0069 (5)	−0.0002 (4)	−0.0010 (4)
O3	0.0157 (6)	0.0253 (6)	0.0072 (5)	−0.0025 (5)	−0.0004 (4)	−0.0001 (4)
O4	0.0244 (7)	0.0162 (6)	0.0299 (8)	−0.0021 (5)	−0.0116 (6)	−0.0034 (5)
O5	0.0256 (7)	0.0168 (6)	0.0221 (7)	−0.0001 (5)	−0.0081 (5)	−0.0025 (5)
O6	0.0301 (8)	0.0153 (7)	0.0541 (11)	0.0024 (6)	−0.0136 (8)	−0.0090 (7)
O7	0.0187 (6)	0.0300 (7)	0.0136 (6)	−0.0096 (5)	−0.0040 (5)	0.0010 (5)
O8	0.0145 (6)	0.0303 (7)	0.0169 (6)	−0.0085 (5)	−0.0045 (5)	0.0055 (5)
O9	0.0146 (6)	0.0271 (7)	0.0223 (7)	−0.0042 (5)	0.0027 (5)	0.0063 (5)
O10	0.0202 (6)	0.0169 (6)	0.0166 (6)	−0.0025 (5)	0.0012 (5)	−0.0002 (5)
O11	0.0180 (6)	0.0176 (6)	0.0190 (6)	−0.0021 (5)	0.0025 (5)	−0.0009 (5)
O12	0.0555 (12)	0.0136 (7)	0.0512 (12)	−0.0006 (7)	0.0156 (9)	−0.0018 (7)
N1	0.0120 (6)	0.0130 (6)	0.0139 (6)	−0.0021 (5)	0.0007 (5)	−0.0016 (5)
N2	0.0129 (6)	0.0171 (7)	0.0090 (6)	−0.0055 (5)	−0.0011 (5)	−0.0020 (5)
N3	0.0130 (6)	0.0172 (7)	0.0094 (6)	−0.0027 (5)	−0.0020 (5)	0.0000 (5)
N4	0.0162 (7)	0.0153 (7)	0.0253 (8)	−0.0028 (6)	−0.0022 (6)	−0.0017 (6)
N5	0.0131 (6)	0.0137 (6)	0.0175 (7)	−0.0027 (5)	−0.0018 (5)	0.0012 (5)
N6	0.0220 (8)	0.0162 (7)	0.0195 (8)	−0.0003 (6)	−0.0005 (6)	−0.0008 (6)
C1	0.0214 (9)	0.0221 (9)	0.0145 (8)	0.0037 (7)	−0.0051 (7)	−0.0047 (7)
C2	0.0222 (9)	0.0225 (9)	0.0153 (8)	0.0028 (7)	−0.0057 (7)	−0.0082 (7)
C3	0.0124 (7)	0.0140 (7)	0.0124 (7)	−0.0043 (6)	0.0002 (6)	−0.0025 (6)
C4	0.0143 (8)	0.0231 (9)	0.0152 (8)	0.0008 (6)	−0.0030 (6)	−0.0071 (6)
C5	0.0151 (8)	0.0233 (9)	0.0154 (8)	−0.0016 (6)	−0.0025 (6)	−0.0085 (6)
C6	0.0155 (8)	0.0158 (7)	0.0139 (8)	−0.0013 (6)	−0.0008 (6)	−0.0032 (6)
C7	0.0167 (8)	0.0142 (7)	0.0141 (8)	−0.0004 (6)	−0.0024 (6)	−0.0009 (6)
C8	0.0120 (7)	0.0153 (7)	0.0105 (7)	−0.0027 (6)	−0.0016 (5)	−0.0007 (5)
C9	0.0157 (8)	0.0148 (7)	0.0121 (7)	−0.0007 (6)	−0.0007 (6)	−0.0016 (6)
C10	0.0163 (8)	0.0153 (7)	0.0124 (7)	−0.0021 (6)	−0.0020 (6)	0.0002 (6)
C11	0.0205 (8)	0.0136 (7)	0.0138 (8)	−0.0039 (6)	−0.0028 (6)	−0.0014 (6)
C12	0.0211 (8)	0.0146 (7)	0.0125 (7)	−0.0064 (6)	−0.0017 (6)	0.0001 (6)
C13	0.0118 (7)	0.0146 (7)	0.0105 (7)	−0.0013 (5)	−0.0017 (5)	0.0000 (5)
C14	0.0278 (9)	0.0140 (8)	0.0134 (8)	−0.0051 (7)	−0.0033 (7)	−0.0012 (6)
C15	0.0295 (10)	0.0168 (8)	0.0133 (8)	−0.0095 (7)	−0.0039 (7)	0.0015 (6)
C16	0.0267 (10)	0.0235 (10)	0.0293 (11)	−0.0009 (8)	−0.0048 (8)	0.0068 (8)
Cl1	0.0292 (2)	0.0199 (2)	0.0288 (2)	−0.00077 (18)	−0.01067 (19)	−0.00114 (17)
Cl2	0.0267 (3)	0.0391 (3)	0.0304 (3)	−0.0049 (2)	−0.0016 (2)	0.0060 (2)

### Geometric parameters (Å, °)

**Table:**

Gd1—O3	2.3216 (13)	C1—H1	0.9500
Gd1—O1	2.3230 (13)	C2—C3	1.395 (2)
Gd1—O2	2.3534 (13)	C2—H2	0.9500
Gd1—O11	2.4601 (13)	C3—C4	1.398 (2)
Gd1—O8	2.4879 (14)	C3—C3^i^	1.481 (3)
Gd1—O7	2.4872 (14)	C4—C5	1.379 (2)
Gd1—O5	2.4958 (14)	C4—H4	0.9500
Gd1—O4	2.4967 (14)	C5—H5	0.9500
Gd1—O10	2.4992 (14)	C6—C7	1.384 (2)
Gd1—N6	2.9021 (17)	C6—H6	0.9500
Gd1—N5	2.9152 (15)	C7—C8	1.394 (2)
Gd1—N4	2.9277 (16)	C7—H7	0.9500
O1—N1	1.3308 (18)	C8—C9	1.396 (2)
O2—N2	1.3346 (18)	C8—C13^ii^	1.479 (2)
O3—N3	1.3310 (18)	C9—C10	1.376 (2)
O4—N4	1.277 (2)	C9—H9	0.9500
O5—N4	1.265 (2)	C10—H10	0.9500
O6—N4	1.219 (2)	C11—C12	1.379 (2)
O7—N5	1.271 (2)	C11—H11	0.9500
O8—N5	1.271 (2)	C12—C13	1.391 (2)
O9—N5	1.217 (2)	C12—H12	0.9500
O10—N6	1.268 (2)	C13—C14	1.394 (2)
O11—N6	1.280 (2)	C13—C8^iii^	1.479 (2)
O12—N6	1.215 (2)	C14—C15	1.374 (3)
N1—C5	1.343 (2)	C14—H14	0.9500
N1—C1	1.347 (2)	C15—H15	0.9500
N2—C6	1.348 (2)	C16—Cl1	1.766 (2)
N2—C10	1.352 (2)	C16—Cl2	1.774 (2)
N3—C11	1.343 (2)	C16—H16A	0.9900
N3—C15	1.349 (2)	C16—H16B	0.9900
C1—C2	1.378 (3)		
			
O3—Gd1—O1	85.06 (5)	C5—N1—C1	121.14 (15)
O3—Gd1—O2	154.22 (5)	O2—N2—C6	119.65 (14)
O1—Gd1—O2	83.54 (5)	O2—N2—C10	119.01 (14)
O3—Gd1—O11	85.96 (5)	C6—N2—C10	121.33 (15)
O1—Gd1—O11	122.79 (4)	O3—N3—C11	120.02 (15)
O2—Gd1—O11	80.97 (5)	O3—N3—C15	118.90 (15)
O3—Gd1—O8	123.47 (4)	C11—N3—C15	121.06 (15)
O1—Gd1—O8	148.53 (5)	O6—N4—O5	122.12 (18)
O2—Gd1—O8	74.82 (4)	O6—N4—O4	122.15 (18)
O11—Gd1—O8	76.46 (5)	O5—N4—O4	115.72 (15)
O3—Gd1—O7	72.31 (4)	O6—N4—Gd1	177.12 (15)
O1—Gd1—O7	150.94 (5)	O5—N4—Gd1	57.84 (9)
O2—Gd1—O7	124.33 (4)	O4—N4—Gd1	57.95 (9)
O11—Gd1—O7	74.51 (5)	O9—N5—O8	122.12 (16)
O8—Gd1—O7	51.32 (4)	O9—N5—O7	122.00 (16)
O3—Gd1—O5	125.67 (5)	O8—N5—O7	115.87 (15)
O1—Gd1—O5	79.17 (5)	O9—N5—Gd1	175.14 (13)
O2—Gd1—O5	74.49 (5)	O8—N5—Gd1	58.03 (9)
O11—Gd1—O5	144.96 (5)	O7—N5—Gd1	58.00 (8)
O8—Gd1—O5	73.25 (5)	O12—N6—O10	122.46 (18)
O7—Gd1—O5	99.15 (5)	O12—N6—O11	121.17 (17)
O3—Gd1—O4	75.01 (5)	O10—N6—O11	116.37 (15)
O1—Gd1—O4	78.88 (5)	O12—N6—Gd1	177.69 (16)
O2—Gd1—O4	124.87 (5)	O10—N6—Gd1	59.06 (9)
O11—Gd1—O4	150.14 (5)	O11—N6—Gd1	57.34 (8)
O8—Gd1—O4	94.89 (5)	N1—C1—C2	120.02 (17)
O7—Gd1—O4	77.84 (5)	N1—C1—H1	120.0
O5—Gd1—O4	51.08 (5)	C2—C1—H1	120.0
O3—Gd1—O10	76.63 (5)	C1—C2—C3	120.99 (16)
O1—Gd1—O10	71.20 (4)	C1—C2—H2	119.5
O2—Gd1—O10	77.83 (5)	C3—C2—H2	119.5
O11—Gd1—O10	51.76 (4)	C2—C3—C4	116.88 (16)
O8—Gd1—O10	124.27 (5)	C2—C3—C3^i^	121.81 (19)
O7—Gd1—O10	118.86 (5)	C4—C3—C3^i^	121.3 (2)
O5—Gd1—O10	141.26 (5)	C5—C4—C3	120.61 (17)
O4—Gd1—O10	140.09 (5)	C5—C4—H4	119.7
O3—Gd1—N6	80.79 (5)	C3—C4—H4	119.7
O1—Gd1—N6	96.87 (5)	N1—C5—C4	120.33 (16)
O2—Gd1—N6	77.73 (5)	N1—C5—H5	119.8
O11—Gd1—N6	25.98 (4)	C4—C5—H5	119.8
O8—Gd1—N6	100.45 (5)	N2—C6—C7	120.24 (16)
O7—Gd1—N6	97.21 (5)	N2—C6—H6	119.9
O5—Gd1—N6	152.20 (5)	C7—C6—H6	119.9
O4—Gd1—N6	155.69 (5)	C6—C7—C8	120.07 (16)
O10—Gd1—N6	25.79 (4)	C6—C7—H7	120.0
O3—Gd1—N5	97.81 (4)	C8—C7—H7	120.0
O1—Gd1—N5	164.46 (4)	C7—C8—C9	117.78 (15)
O2—Gd1—N5	99.36 (4)	C7—C8—C13^ii^	123.06 (15)
O11—Gd1—N5	72.72 (4)	C9—C8—C13^ii^	119.14 (15)
O8—Gd1—N5	25.68 (4)	C10—C9—C8	120.69 (16)
O7—Gd1—N5	25.68 (4)	C10—C9—H9	119.7
O5—Gd1—N5	86.86 (5)	C8—C9—H9	119.7
O4—Gd1—N5	87.08 (5)	N2—C10—C9	119.89 (16)
O10—Gd1—N5	124.33 (4)	N2—C10—H10	120.1
N6—Gd1—N5	98.67 (5)	C9—C10—H10	120.1
O3—Gd1—N4	100.41 (5)	N3—C11—C12	120.02 (16)
O1—Gd1—N4	77.11 (5)	N3—C11—H11	120.0
O2—Gd1—N4	99.46 (5)	C12—C11—H11	120.0
O11—Gd1—N4	159.79 (5)	C11—C12—C13	120.29 (16)
O8—Gd1—N4	84.12 (5)	C11—C12—H12	119.9
O7—Gd1—N4	89.07 (5)	C13—C12—H12	119.9
O5—Gd1—N4	25.41 (5)	C12—C13—C14	118.11 (15)
O4—Gd1—N4	25.69 (5)	C12—C13—C8^iii^	120.50 (15)
O10—Gd1—N4	148.30 (5)	C14—C13—C8^iii^	121.35 (15)
N6—Gd1—N4	173.67 (5)	C15—C14—C13	119.68 (16)
N5—Gd1—N4	87.36 (4)	C15—C14—H14	120.2
N1—O1—Gd1	129.39 (10)	C13—C14—H14	120.2
N2—O2—Gd1	124.82 (10)	N3—C15—C14	120.67 (17)
N3—O3—Gd1	127.48 (10)	N3—C15—H15	119.7
N4—O4—Gd1	96.36 (10)	C14—C15—H15	119.7
N4—O5—Gd1	96.75 (11)	Cl1—C16—Cl2	111.26 (12)
N5—O7—Gd1	96.32 (10)	Cl1—C16—H16A	109.4
N5—O8—Gd1	96.29 (10)	Cl2—C16—H16A	109.4
N6—O10—Gd1	95.16 (11)	Cl1—C16—H16B	109.4
N6—O11—Gd1	96.67 (10)	Cl2—C16—H16B	109.4
O1—N1—C5	119.58 (14)	H16A—C16—H16B	108.0
O1—N1—C1	119.26 (15)		

Symmetry codes: (i) −*x*+3, −*y*+1, −*z*+1; (ii) *x*, *y*, *z*+1; (iii) *x*, *y*, *z*−1.

### Hydrogen-bond geometry (Å, °)

**Table:**

*D*—H···*A*	*D*—H	H···*A*	*D*···*A*	*D*—H···*A*
C5—H5···O7^iv^	0.95	2.41	3.082 (2)	127.
C9—H9···O9^v^	0.95	2.57	3.287 (2)	132.
C12—H12···O2^vi^	0.95	2.43	3.300 (2)	152.
C16—H16B···O12^v^	0.99	2.43	3.246 (3)	139.
C16—H16A···O8	0.99	2.56	3.302 (3)	132.
C16—H16A···O9	0.99	2.50	3.084 (3)	117.

Symmetry codes: (iv) *x*+1, *y*, *z*; (v) −*x*+1, −*y*+2, −*z*+2; (vi) −*x*+2, −*y*+2, −*z*+1.
